# Medication management information priorities of people living with dementia and their carers: a scoping review

**DOI:** 10.1093/ageing/afae200

**Published:** 2024-09-17

**Authors:** Alexander J Clough, Danijela Gnjidic, Amanda J Cross, Natali Jokanovic, Karen Watson, Jacqueline Wesson, Stephanie Beshara, Justin Cheng, Mouna J Sawan

**Affiliations:** Sydney Pharmacy School, Faculty of Medicine and Health, University of Sydney, Camperdown, NSW, Australia; Sydney Pharmacy School, Faculty of Medicine and Health, University of Sydney, Camperdown, NSW, Australia; Centre for Medicine Use and Safety, Faculty of Pharmacy and Pharmaceutical Science, Monash University, Parkville, VIC, Australia; Department of Infectious Diseases, The Alfred Hospital and Central Clinical School, Monash University, Melbourne, VIC, Australia; Sydney Nursing School, Faculty of Medicine and Health, University of Sydney, Camperdown, NSW, Australia; School of Health Sciences, Faculty of Medicine and Health, University of Sydney, Camperdown, NSW, Australia; Sydney Pharmacy School, Faculty of Medicine and Health, University of Sydney, Camperdown, NSW, Australia; Sydney Pharmacy School, Faculty of Medicine and Health, University of Sydney, Camperdown, NSW, Australia; Sydney Pharmacy School, Faculty of Medicine and Health, University of Sydney, Camperdown, NSW, Australia

**Keywords:** scoping review, medication management, dementia, caregivers information needs, older people

## Abstract

**Background:**

People living with dementia and their carers often experience difficulties in effectively managing medications and have indicated they lack necessary support, information and guidance. Recognising the medication management information needs of this population is an important first step in addressing these issues.

**Objectives:**

To identify the priorities for information on medication management expressed by people living with dementia and their carers.

**Methods:**

A scoping review with systematic search was conducted from inception to 12 May 2023 for any original studies that reported the information needs of people living with dementia and their carers (informal, i.e. unpaid or within an existing relationship) regarding medication management. Two authors independently screened the abstracts, full-texts and extracted data. Study characteristics were described descriptively, and themes of information need were extracted using an iterative approach.

**Results:**

Of the 11 367 records screened, 35 full-texts were included. All studies (n = 35) involved carers, with 17 also including people living with dementia. Most studies (n = 30) were conducted in the community and used qualitative methods (n = 32). Five major themes of information need were identified: critical medication information; medication effects; medication indication(s); disease progression and impact on medications; and safe and appropriate administration of medications. People living with dementia and their carers indicated they need more medication management information generally and want it simple, tailored and relevant.

**Conclusions:**

This review highlights the key medication information priorities for people living with dementia and their carers and will help guide the provision of medication management guidance and development of new information resources.

## Key Points

- First review identifying the medication management information needs reported by people living with dementia and their carers.
- More information is required on medication indications, effects, duration, ideal administration, and rationales for ongoing use.
- People living with dementia and their carers should be involved in discussions about their medications to promote safe use.

## Introduction

People living with dementia have more comorbidities than people without dementia leading to higher rates of medication use, including potentially inappropriate medications and polypharmacy [1, 2]. These patterns of medication use can make managing these medication regimens very challenging, resulting in higher stress and burden for people living with dementia and their carers [3, 4]. These factors, alongside cognitive decline related to dementia, impede the ability of people living with dementia and their carers to perform medication management, increasing the risk of medication-related adverse events [5]. Medication management encompasses any one of the following activities: selecting, supplying, preparing, administering, recording, monitoring and reviewing medications; and is a continual process from the initial prescribing of a medication through to ongoing management and review [6]. People living with dementia commonly manage their own medications in the early stages of dementia before their carers take a more prominent role as the disease progresses [7]. Carers have reported their involvement in every medication management activity, most commonly in administering, selecting, ordering and collecting medications [8].

Both people living with dementia and their carers have expressed unique difficulties with these activities, with people living with dementia often viewed as passive recipients of care and unable to manage their own health [9]. Combatting this by improving the self-efficacy of people living with dementia in caring for themselves has demonstrated increases in quality of life [7, 10]. Additionally, evidence-based interventions that educate both the person living with dementia and their carer, and using person-centred care have improved transitions of care [11]. Empowering and educating people living with dementia and their carers about medications can thus help with medication management and improve health outcomes, with carers reporting increased confidence and satisfaction when receiving better-perceived and understandable guidance [12].

While there are informational materials available, a recent scoping review and environmental scan found that no current resources provide guidance on all aspects of medication management that are readable, practical and co-developed with people living with dementia and their carers [13]. Additionally, although the care needs of older adults in general have been explored regarding medication management at transitions of care, previous reviews have not explored the perspectives of people living with dementia [14]. Successful interventions depend upon a strong evidence base that considers the experiences and views of people receiving care, in this case people living with dementia and their carers [11]. To address this gap, the aim of this scoping review is to identify the priorities for medication management information expressed by people living with dementia and carers in published studies.

## Methods

This scoping review was developed following the Joanna Briggs Institute (JBI) methodology for scoping reviews [15] and the protocol was registered on Open Science Framework (https://osf.io/7xdmc). The Preferred Reporting Items for Systematic Review and Meta-Analysis extension for scoping reviews (PRISMA-ScR) was followed (see Appendix 1 in the Supplementary Data for the checklist) [16].

### Search strategy

The search strategy was modified from a previous scoping review that explored the experiences, priorities and perceptions of carers of people living with dementia in residential aged care facilities (RACFs) by removing terms related to RACFs and including terms related to medications and health information [17]. A University of Sydney librarian was consulted on the search strategy before running the final search. A systematic search was then conducted in seven electronic databases (Medline [Ovid], Embase [Ovid], Cochrane Library [Wiley], PsycINFO [Ovid], Web of Science [Clarivate], CINAHL [Ebsco] and Ageline [Ebsco]) from inception up to 12 May 2023. Key search terms were related to ‘dementia’, ‘identifying priorities’, ‘information’ and ‘medications’. The search strategy, including all identified keywords and index terms, was adapted for each included database (see Appendix 2 in the Supplementary data for the search strategy in Medline).

Identified studies were exported to EndNote (EndNote X9, Berkley, California, United States of America) and Covidence systematic review software (Veritas Health Innovation, Melbourne, Australia) and duplicates removed in Covidence.

### Study eligibility

Included studies described the medication management information needs reported only by people living with dementia and/or carers of people living with dementia. All types of dementia, study setting and design and medications and medication regimes were considered. For this review, carers were defined as informal (unpaid) carers or carers who provided care within the context of an existing relationship (e.g. family or friend) [18]. Studies were excluded if the information needs were reported by any other population (such as healthcare professionals), or the information needs did not relate to medication management. Studies were also excluded if no full-text was available, they were not in English, and if they were reviews or not original studies.

### Study screening and selection

Two authors independently screened the titles and abstracts, and full-texts of all identified studies (two of: AJCl, AJCr, NJ, KW, JW, SB, JC, MJS). Discrepancies were resolved by a third author (DG). Forwards and backwards citation tracking was used to identify any additional studies.

### Data extraction

Data extraction for each study was completed by two authors independently (AJCl and AJCr, NJ, KW, JW, SB, JC or MJS). Study characteristics, methods and aims and focus regarding medications (general, a specific class or medication, or pattern of medication use) and data on the specific aspects of medication management reported were extracted. Pertinent quotes reported in the studies were identified and extracted.

The identified information needs, and experiences with medication management, as they relate to the four dimensions of health information processing defined by Sørensen et al. were also extracted [19]. These four dimensions are the ability of someone to: (i) access medication management information; (ii) understand medication management information; (iii) interpret and evaluate medication management information; and (iv) make informed decisions on medications and comply with medication advice. This was so the identified needs could be described according to how people living with dementia and their carers genuinely use information.

### Analysis and presentation of results

The data on study characteristics and the aspect(s) of medication management reported in each study were described descriptively and tabulated to identify the most commonly identified areas of need. The content analysis of medication management information needs was performed using an iterative approach. The information needs were first listed verbatim as reported in the studies and then sorted into the aspect of medication management they aligned with. Codes were then developed from the results based on content (specific need and quotes) and who reported the need (people living with dementia and/or their carers). The codes were refined by comparing the similarities and differences between codes and emerging themes were extracted relating to the medication management information reported.

Two authors (AJCl and MJS) independently coded the extracted data and compared interpretations to ensure reliability. Theme development was discussed at project team meetings and iteratively refined based on group consensus. The themes were finally sorted according to how many studies reported those themes.

The extracted information needs defined by Sørensen et al. were next classified according to how people living with dementia and their carers accessed, understood, interpreted and actioned medication management information [19]. The dimensions were also used to explore what people living with dementia and their carers found both helpful and challenging when receiving and using medication management information.

## Results

### Search results

After duplicate removal, 11 516 titles and abstracts of studies were screened, 149 full-texts were assessed for eligibility and 35 studies were included in the final analysis (see Appendix 3 in the Supplementary Data for the PRISMA flowchart) [20–54]. The main reasons for study exclusion were the studies: not reporting medication management information needs (n = 49); no full-text available (n = 38); and not being original studies (n = 18).

**Fig. 1:**
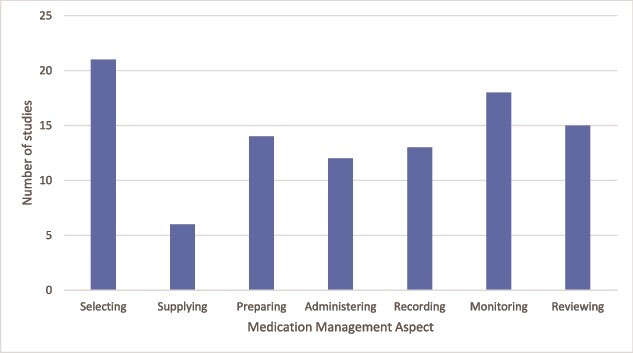
Number of studies reporting information needs reported by people living with dementia and their carers by aspect of medication management.

The full characteristics of included studies and aspects of medication management reported are described in Table 1. Most studies were conducted in the community (n = 30), with four studies also recruiting people living with dementia who resided in RACFs. Most studies (n = 23) used qualitative semi-structured interviews to explore the information priorities and six studies used focus groups. All studies recruited carers, with 17 studies also recruiting people living with dementia. The most reported aspects of medication management needs were selecting (n = 21), monitoring (n = 18) and reviewing (n = 15) medications (Fig. 1). Most studies explored medication management of all prescribed medications (n = 29), with three studies focussing on specific drug classes (antipsychotics, [n = 2] and dementia-specific [n = 1]) and the remaining three on other patterns of medication use.

**Table 1:** Characteristics of identified studies

*Author, year*	*Country*	*Healthcare setting*	*Study aims*	*Methods*	*Medication focus*	*Population (n)*	*Aspects of medication management reported*
Alsaeed, 2021 [20]	UK	Community & RACFs	Identify and examine medication-related problems for people with dementia and their carers.	Qualitative, semi-structured interviews	General	People with dementia (10) & carers (11)	a, c, d, e, f
Armstrong, 2021 [21]	USA	Dementia clinic	Discover helpful care aspects and unmet needs, particularly carer, of people with dementia with Lewy bodies.	Qualitative, semi-structured interviews	General	People with dementia (20) & carers (25)	b, f, g
Badawoud, 2023 [22]	Saudi Arabia	Community	Determine the carer burden of people with Alzheimer’s disease.	Cross-sectional survey.	General	Carers (148)	a, c, d, e, f
Bardach, 2021 [23]	USA	Community & RACFs	Identify the scope of dementia-related knowledge gaps.	Qualitative, semi-structured interviews	General	Carers (294)	a, b, e, f, g
Barry, 2021 [24]	UK	Community	Explore the perspectives of carers and people with dementia about medication management.	Qualitative, semi-structured interviews	General	People with dementia (18) & carers (15)	a
Behrman, 2017 [25]	UK	Community	Define and understand safety regarding community healthcare of people with dementia.	Qualitative, semi-structured interviews	General	Carers (10)	c
Bloomstone, 2020 [26]	USA	Community	Evaluate educational materials about prescribing cascades in people with Alzheimer’s disease.	Qualitative, semi-structured interviews	Prescribing cascades	People with dementia (12) & carers (14)	e, f, g
Cornege-Blokland, 2012 [27]	Netherlands	RACFs	Understand the role of carers in the decision-making process preceding an antipsychotic prescription.	Qualitative, semi-structured interviews	Antipsychotics	Carers (37)	f
De Bellis, 2017 [28]	Australia	RACFs	Explore experience, knowledge and perceptions of challenging behavioural and psychological symptoms of dementia and associated antipsychotic use.	Qualitative, semi-structured interviews	Antipsychotics	Carers (6)	a, d, f, g
Deeks, 2016 [29]	Australia	Community & care transitions	Explore medication processes that occur during acute care episodes and in care transitions for people with dementia.	Qualitative, semi-structured interviews	General	Carers (4)	g
El-Saifi, 2019 [30]	Australia	Community	Better understand the determinants of medication non-adherence in carers of people with dementia.	Qualitative, semi-structured interviews	General	Carers (20)	a, b, c, d, e, f
El-Saifi, 2021 [31]	Australia	Community	Report carer’s perceptions about the role of community pharmacists.	Qualitative, semi-structured interviews	General	Carers (20)*	a, b, c, d, f, g
Gillespie, 2015 [32]	Australia	Community	Explore the views of ethnic minority family carers of people with dementia and their medication management experiences.	Qualitative, semi-structured interviews	General	Carers (29)	a, b, c, d, f, g
Green, 2020 [33]	USA	Community	Explore people with dementia, carers and physician perspectives on deprescribing and recommended language for deprescribing discussions to inform an intervention increasing deprescribing awareness.	Qualitative, semi-structured interviews	Deprescribing	People with dementia (17) & carers (16)	g
Horne, 2018 [34]	Australia	RACFs	Evaluate the usefulness of a web-based medication management information resource.	Focus groups	General	Carers (16)	d, g
Kimzey, 2022 [35]	USA	Community	Explore the development of health literacy competencies among people with dementia and their carers.	Focus groups	General	People with dementia (15) & carers (28)	a, c, e, f
Lynnerup, 2023 [36]	Denmark	Community	Explore perspectives on medication safety from older migrants with cognitive impairment using five or more medications daily and their close relatives.	Qualitative, semi-structured interviews	General	People with dementia (8) & carers (9)	a, c, e, f, g
Maidment, 2017 [37]	UK	Community	Describe and understand the key medication challenges experienced by people with dementia and the potential role of pharmacists.	Focus groups	General	People with dementia (4) & carers (11)	c, d, e, g
Martinez-Lage 2011 [38]	USA, France, Germany & Spain	Community	Investigate experiences and perceptions of carers of people with dementia using transdermal patch therapy.	Qualitative, semi-structured interviews and quantitative interviews	Rivastigmine	Carers (206)	a, g
McCloskey, 2018 [39]	UK	Community & RACFs	Explore proxy decision makers’ expectations of medications for people with advanced dementia and consider how they change with changing care goals and dementia progression.	Qualitative, semi-structured interviews	General	Carers (15)	e, f, g
Poland, 2014 [40]	UK	Community	Describe the Public Patient Involvement process to inform and validate the development of a future research proposal.	Adapted focus groups	General	Carers (9)	a, c, d, f, g
Rathnayake, 2020 [41]	Australia	Community	Examine needs of carers of people with dementia concerning the management of functional disability of people with dementia, carer burden and use of mHealth applications when seeking health information.	Quantitative survey	General	Carers (166)	d
Rees, 2020 [42]	UK	Community	Explore how the self-management of long-term comorbidities is experienced and negotiated by people with dementia and carers.	Qualitative, semi-structured interviews	General	People with dementia (11) & carers (22)	d
Reeve, 2023 [43]	Australia	Community	Determine what questions about medicine use are important to people with dementia and their carers and if the questions have been answered by research.	Qualitative survey	General	People with dementia (14) & carers (38)	a, b, c, d, e, f, g
Sawan, 2021 [44]	Australia	Hospital discharge	Explore the experiences and perspectives of carers about the medication management guidance provided at hospital discharge.	Qualitative, semi-structured interviews	General	Carers (31)	a, e, f, g
Seike, 2014 [45]	Japan	Community	Examine the learning needs and post-learning attitude changes of people with dementia and their families to assess the effectiveness of an interdisciplinary educational support program.	Quantitative survey	General	People with dementia (51) & carers (119)	a
Shariff, 2020 [46]	UK	Community & RACFs	Investigate the characteristics of oral solid dosage forms that contribute to age appropriate dosage design.	Qualitative, semi-structured interviews	Oral solid dosage	People with dementia (>0) carers (7)	c
Smith, 2015 [47]	UK	Community	Examine the experiences of carers when providing medication-related assistance for a person with dementia and indicate how services could be more responsive to the needs of carers.	Qualitative, semi-structured interviews	General	People with dementia (5) & carers (14)	a, c, e, f
Wald, 2003 [48]	UK	Community	Determine and prioritise what information dementia carers wish to know at the time of and after diagnosis and in what form the information should be presented.	Qualitative, structured interviews	General	Carers (100)	a
Wherton, 2008 [49]	UK	Community & RACFs	Identify the daily activities of people with dementia living at home that could be supported by technology.	Qualitative, semi-structured interviews	General	People with dementia (8) & carers (10)	d
While, 2013 [50]	Australia	Community	Examine the perspectives of people with dementia and carers in their medication management experience compared to older adults without dementia.	Qualitative, semi-structured interviews	General	People with dementia (8) & carers (9)	a, b, c, d, e, f
Wolfs, 2010 [51]	Netherlands	Community	Investigate to what extent and in what way people with dementia utilise available treatment options and identify factors and reasons that play a role in non-utilisation.	Qualitative, semi-structured interviews	General	People with dementia (177) & carers (252)	a
Wolverson, 2023 [52]	UK	Mental health ward	Establish the information needs of people with dementia and their family when admitted to a mental health ward and explore if existing ward information leaflets meet the information needs.	Focus groups and content analysis	General	People with dementia (1–5) carers (1–5)**	a
Yeh, 2021 [53]	USA	Community	Assess satisfaction with and elicit recommendations for improving end-of-life experiences of people with dementia.	Cross-sectional survey	General	Carers (53)	a, f
Zubatsky, 2016 [54]	USA	Community	Explore the challenges that first-time carers encountered with health care team members at diagnosis.	Focus groups	General	Carers (13)	a, c, f

*Aspects of medication management reported: selection^a^, supplying^b^, preparation^c^, administration^d^, recording^e^, monitoring^f^, reviewing^g^*

*UK: United Kingdom; USA: United States of America; RACF: Residential aged care facilities*

*Same population as El-Saifi, 2019 [30] **Six focus group participants, no breakdown given of number of people with dementia and carers.

### Medication management information priorities

Five main themes, reported in 25% or more studies, were identified as priorities for medication management information and are discussed below according to the frequency of studies reporting each theme.

#### Critical medication information (n = 23 studies, 65.7%)

The most reported area of information need was related to critical medication information, which was often described as lacking, missing, or not useful [20, 22–25, 30–32, 35, 36, 38–41, 43–45, 47, 48, 50, 51, 53, 54]. The critical medication information is derived from Australian standards of required information on medication product labels and recommendations to healthcare professionals regarding information provision to patients [55, 56]. It encompasses: active ingredients [50]; medication purpose and warnings [39, 40, 43, 44]; directions for use [22–25, 30, 31, 36, 41, 44, 47, 54]; and the availability of medication options and formulations [20, 30, 32, 35, 38, 45, 48, 51, 53]. As an example, due to allergies, before accepting any new medication a person living with dementia always had to ask ‘how many are there with penicillin in them’ [While, et al., page 739 [50]].

#### The effects of the medications (n = 17 studies, 48.6%)

Next, it was reported there was not enough information on the effects of medications, both in terms of intended benefits and side-effects [20–22, 26–28, 30–32, 36, 39, 40, 43, 44, 47, 50, 54]. Carers drew particular attention to the need for information on the side-effects of sedating medications and antipsychotics, knowing that these medications came with certain risks but not exactly what they were and how they could be managed [21, 28].

“It helps calm them down where they’re not frightened and they’re tolerable, but what’s it doing to them physically? You know, is it taking away from their life?”

[carer in Amstrong, et al., page 6 [21]].

Furthermore, carers wanted information on newly prescribed medications and dementia-specific medications and expressed uncertainty on the effects of these medications, partly because it was not always clear what would have happened if they were not taken [39, 47, 54].

‘The consultant told me that memantine would slow the dementia down … it is difficult to say whether it has slowed it down or not because I can’t tell what the dementia would be like if he wasn’t taking it, you know.’

[carer in McCloskey, et al., page 1118 [39]].

Carers suggested that a checklist with information on the known intended effects and side-effects could assist them in monitoring the medications [40].

#### Why the medication has been prescribed (n = 14 studies, 40.0%)

People living with dementia and their carers frequently reported they do not receive information on the indication(s) for each medication [20, 22, 24, 28, 30–32, 35, 36, 43, 44, 47, 50]. As demonstrated by one person living with dementia: “All I know is they are all tablets… I don’t know what they’re for” [Barry, et al., page 887 [24]].

In cases where information was provided, it was reported that the information was either insufficient and too generic to be useful, or too complex to be fully understood [30]. Complexity was especially common in written information, where medication information sheets and leaflets were too lengthy and information-dense [31].

‘Now that’s one of the difficulties with some medication information packages that you get … they are so long because they are so complex and written in technical terminology, most carers won’t read them…’.

[carer in El-Saifi, et al. (2021), page 467 [31]].

Some carers were concerned that because the person living with dementia did not know what each medication was for, they would selectively take only those they believed to be providing benefit [32].

#### Disease progression and ongoing appropriateness of medications (n = 14 studies, 40.0%)

It was further reported that more information is needed on how the progression of dementia could potentially impact the long-term appropriateness of medications and how to seek assistance on the ongoing management of medications [20, 21, 23, 26, 28, 31–35, 37, 40, 43, 44]. Carers reported they wanted to know the impact of medications on the person they cared for at different stages of the condition to distinguish whether any symptoms or behaviour changes may be due to overprescribing, under-prescribing, the actual condition or something else entirely [21, 23, 28, 32, 35].

‘It’s also hard to know at each step, is it happening because of the disease or is she medicated?... Is it being so relaxed that you haven’t got control of your bowels or is it the disease? I don’t know.’

[carer in De Bellis, et al., page 27 [28]].

Four studies reported that people living with dementia and their carers had concerns with the medication regimen due to either the number of medications, or a particular prescription, but did not have enough information to ask for a medication review or approach the topic of deprescribing [31, 33, 37, 43]. They proposed using educational materials to prompt these conversations as:

‘It helps people bring up the subject if they don’t know how.’

[person living with dementia in Green, et al., page 3559 [33]].

#### Safe and appropriate administration of medications (n = 13 studies, 37.1%)

Finally, people living with dementia and their carers reported they needed more information on how to safely and appropriately administer medications [20, 22, 30–32, 34, 37, 40, 41, 43, 46, 50]. This was a theme mostly reported by carers, encompassing timing and method of administration [20, 22, 30–32, 34, 37, 40, 41, 43, 46]; providing clear information to the person living with dementia to aid acceptance of medications [20, 31, 40, 43, 50]; and managing direct administration concerns such as refusal and swallowing difficulties [20, 31, 32, 40, 43]. People living with dementia also reported wanting information on how to safely take their medications [43, 50].

Carers commented that being able to explain why a medication had been prescribed and giving clear instructions for usage helped reduce refusal [20, 31, 40, 50]. However, carers also remarked that guidance was not always applicable to their situation and not address the realities and difficulties in administering medications, such as knowing if and when eye drops had been taken [40].

‘Don’t forget that the clinician and pharmacist can have little or no understanding of the practicalities [of administering medications].’

[carer in Poland, et al., page 3 [40]].

**Fig. 2:**
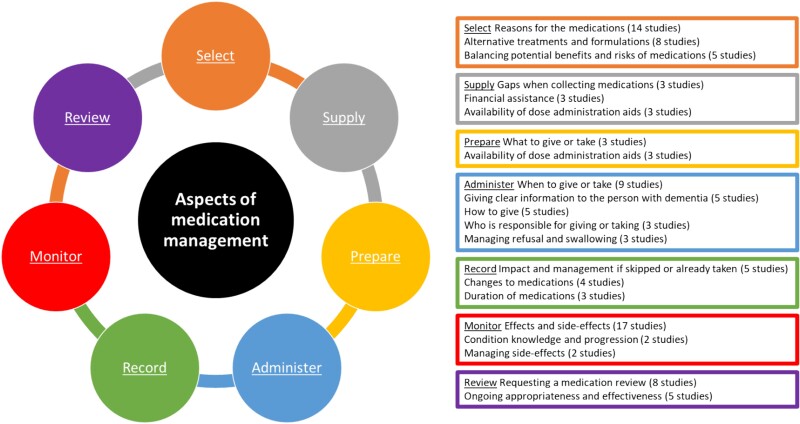
Common themes of medication management information needs identified by people living with dementia and their carers by aspect of medication management.

Additional identified themes not discussed above are presented in Fig. 2 and classified according to the aspect of medication management each theme corresponds to.

### Processing medication management information

Described below are people living with dementia and their carers’ common experiences with receiving and using health information within Sørensen’s four dimensions of health information processing. A summary of the enablers and challenges reported by people living with dementia and their carers of these different dimensions are described in Table 2.

**Table 2:** Enablers and barriers in processing medication management information experienced by people living with dementia and their carers

*Process*	*Enablers*	*Barriers*
Accessing	Pharmacists can be useful in providing information [37]. Internet and written sources good for supplementing healthcare professional information [24, 28, 30, 32, 33, 35, 41, 44, 47, 54]. Support networks provide unique insights for carers [32, 35, 44].	Language difficulties, including too high level and translation requirements [32, 36]. Fragmentation and complexity of care resulting in information gaps [25, 35]. Transportation difficulties to consultations not allowing carers to receive information [50].
Understanding	Simple, relevant information using practical examples helps understanding [26, 34, 43, 52]. Healthcare professional taking time to explain the information to both person living with dementia and their carer [20, 37]. Dose administration aids including Webster-paks are useful in knowing what and when to give/take medications [30].	Written information often complex, long, not appropriate and appears to be targeted to professionals [30, 31, 40, 44]. When not present at the meeting, carers not given opportunity to understand information fully [45, 47]. Information given at transitions of care, when care is fragmented, at the initial diagnosis, and if given all-at-once is difficult to fully understand [38, 44, 47, 48, 50, 52, 53].
Implementing	Brief, one-on-one information for clarity and full understanding [30, 31, 37, 40, 48]. Simple written resources, such as checklists [40]. Medications lists at hospital discharge provide information not otherwise given [44].	Information on benefits and risks of medications too generic [26, 30, 31]. Healthcare professionals and other resources not considering potential prior knowledge of people [52]. Mixing brand and generic names of medications adds confusion [50]
Actioning	Unique, personalised strategies developed over time to manage medications [24, 28, 30, 35]. Question prompts and other ‘empowerment’ tools prompt shared decision-making [26]. Automatic dispensers and personalised medication records useful [30, 34, 50]	Lack of knowledge impairs decision-making on medications [39, 40]. Cold and clinical information, especially received at home, increases anxiety [33, 52]. Complex regimes limit effective proper usage [50]

### Accessing medication information

People living with dementia and their carers wanted and expected, to access medication management information directly from healthcare professionals stating they placed great trust in them [20, 24, 30–32, 35, 39, 44, 48, 50]. However, it was commonly reported that healthcare professionals did not always provide this information, and, if they did, it was insubstantial, lacking relevancy and resulted in recipients having to source their own information [20, 31, 35, 38, 39, 44, 54]. This mostly came from internet searches [31, 38, 44, 54] and printed sources [44, 54] that may be inaccurate or misleading, such treatment duration varying between online suggestions and prescriber recommendations [44]. Additional medication management information was accessed from: patient information sheets [24, 44, 50], direct-to-consumer educational brochures [33, 44], factsheets from dementia organisations [28, 48, 54], support groups [28, 30, 32, 35, 44], family members [36], other healthcare professionals [44, 47], conferences [28, 35] and internet-based resources [24, 28, 30–32, 35, 38, 41, 44, 47, 48, 54].

### Understanding medication information

Many people indicated that written information, while useful, was often difficult to fully understand due to its length and complexity [30, 31, 40, 44]. Thus, it was appreciated when healthcare professionals took the time to not only explain the information they were giving, but also go through written information otherwise accessed [26, 34, 43, 52]. This was considered especially important when there were cultural and religious factors that may affect medication use [20, 37]. Sometimes, carers had to seek healthcare professionals who spoke their language, even if it delayed care [32]. Dose administration aids were commonly used but limitations were noted regarding their practicality for use in this population, such as Webster-paks having small font size [30].

### Interpreting medication information

Frustration at the lack of opportunities and resources to fully interpret and evaluate the medication management information received from healthcare professionals was reported as an issue [26, 30, 31, 35, 47, 54]. People living with dementia and their carers suggested a person-to-person approach providing brief, tailored information as the need arises would be more useful and allow for informed choices to be made [43]. Carers recommended written resources, such as checklists, that could contain key, simple information on medications and assume a certain prior knowledge of the healthcare system [40, 52]. Reputable sources, including the Mayo clinic and dementia websites, were considered valuable by those conducting their own searches, and people living with dementia and their carers commonly triangulated the information to satisfy their requirements [34, 35].

### Actioning medication information

Confidence and the ability to employ useful medication management strategies such as implementing dosing schedules increased in line with experience but, importantly, it also rose when people living with dementia and their carers were involved in shared decision-making [28, 35]. However, when information was not fully given, or carers were not afforded the opportunity to participate in shared decision-making, problems arose [40]. This sometimes resulted in medication decisions being made without expert advice, such as carers making up their own medications lists, increasing the potential for errors and carer burden [40, 44]. Medication reviews were also seen as great facilitators in improving medication management [31, 32].

## Discussion

To our knowledge, this is the first review of medication management information needs and priorities of people living with dementia and their carers, involving 35 studies with 378 people living with dementia and 1757 carers. It was reported that more information was required on every aspect of medication management to ensure the safe and appropriate use of medications. The priorities for information need related to critical medical information, the reasons for and effects of medications, impact of dementia progression on the use of medications, and safe and appropriate administration of medications. People living with dementia and their carers wanted clear, concise and relevant information and to be more involved in medication decisions. This review identified that people living with dementia and their carers are provided limited and inadequate information to manage medications.

Healthcare professionals were seen as both significant supporters for people living with dementia and their carers but also imposing barriers for receiving, understanding and putting medication management information into action [35]. These findings align with other studies, where people living with dementia and their carers expressed dissatisfaction with the communication from healthcare professionals [17, 57], perceiving it as disjointed and not meeting their needs [13, 58]. Transitions of care were described as particularly challenging periods for managing medications, primarily due to fragmented care and a lack of consideration for the impact of dementia on medication management [29, 44]. From a carer perspective, this exacerbates their stress and burden which worsens their health and can subsequently negatively impact the person they are caring for [59]. Current planning misses these unique challenges and does not address the realities, such as the importance of the carer and managing changed behaviour during goals of care discussions [11]. Indeed, a previous review of successful care transition interventions for medication continuity among older adults did not describe what impact any level of cognitive impairment would have upon the ‘success’ of care transitions [14]. Proposed solutions could include incorporating a multidisciplinary team as part of in-hospital care for people living with dementia, such as involving pharmacists and pharmacologists in physician rounds to detect potential medication issues and propose interventions acceptable to patients, carers and prescribers [60, 61]. Fundamentally, prioritising person-centred care, involving the person living with dementia and their carer in medication-related decisions while considering their acquired experience and knowledge, and having coordinated, responsive and tailored care can help address medication management challenges [11, 35, 43, 52, 62].

Not only is emphasising patient-centred care preferable to people living with dementia and their carers [63, 64], but it has also demonstrated improved health outcomes [65]. Carers specifically highlighted the benefit of one-on-one meetings with a healthcare professional in which information was briefly and simply explained and repeated to ensure full understanding [30, 31, 37]. This verbal communication should be combined with accurate and understandable written information, due to both people living with dementia and their carers’ needs [26, 40], and because of the frequent use and reliance of written information [58]. Developing these materials requires a collaborative approach between people living with dementia, carers, healthcare professionals and researchers that emphasises experience-gathering and co-design at every stage of resource development [66]. While previous reviews have explored the issues related medication management experienced by people living with dementia and their carers, and examined the available resources to assist this, none identified what the specific needs are [13, 67]. This review adds to the evidence by identifying the information needs of people living with dementia and their carers regarding medication management so these can be addressed in any future resource development.

Further work is required to directly confirm the information gaps identified in this review with people living with dementia, carers and healthcare professionals and explore the priorities for medication management information across care settings that have not been captured in this scoping review. The identified priorities can be used as foundations for the development of co-designed medication management tools that address the unique needs of people living with dementia and carers.

### Strengths and limitations

This review has several strengths. First, several databases were searched to identify studies reporting medication management information needs of people living with dementia and their carers. Second, data integrity was assured with two authors reviewing and extracting data for each study. Third, reference list checking and citation tracking ensured any studies missed through initial searches were captured.

This review does have one major limitation, however. Although the number of studies reporting each information need was extracted, the ordered priorities and how many people within each study reported the information need was not extracted. This is because not all studies included this data, and although direct participant quotes were extracted, they may not accurately reflect the priority of that need. Further research is required to confirm the information gaps identified in this review and establish the priorities of these needs. Once validated, these information priorities may act as the foundations of co-designed resources and tools providing accurate, targeted and understandable medication management guidance.

## Conclusions

This is the first scoping review to explore and identify the medication management information needs of people living with dementia and their carers. We found that increased, tailored, well-communicated information from healthcare professionals is required. Healthcare professionals are recognized as important sources of this information and people living with dementia and their carers clearly expressed a desire to be more involved in decision-making processes. Written resources need to be more comprehensive and understandable to people living with dementia and their carers that directly address their information needs. Future studies should further confirm the identified information needs to develop targeted medication management information resources.

## Supplementary Material

aa-24-0561-File002_afae200
