# Supplementary material for: Medication management information priorities of people living with dementia and their carers: a scoping review
Source: Age Ageing. 2024 Sep 17;53(9):afae200. doi: 10.1093/ageing/afae200 (PMC11406060; doi:10.1093/ageing/afae200)
Supplement: aa-24-0561-File002_afae200 [file aa-24-0561-file002_afae200.docx]

**Supplementary D**

**Appendix 1: Preferred Reporting Items for Systematic reviews and Meta-Analyses extension for Scoping Reviews (PRISMA-ScR) Checklist**

| **SECTION** | **ITEM** | **PRISMA-ScR CHECKLIST ITEM** | **REPORTED**  **ON PAGE #** |
| --- | --- | --- | --- |
| **TITLE** | | | |
| Title | 1 | Identify the report as a scoping review. | 1 |
| **ABSTRACT** | | | |
| Structured summary | 2 | Provide a structured summary that includes (as applicable): background, objectives, eligibility criteria, sources of evidence, charting methods, results, and  conclusions that relate to the review questions and objectives. | 1 |
| **INTRODUCTION** | | | |
| Rationale | 3 | Describe the rationale for the review in the context of what is already known. Explain why the review  questions/objectives lend themselves to a scoping review approach. | 2 |
| Objectives | 4 | Provide an explicit statement of the questions and objectives being addressed with reference to their key elements (e.g., population or participants, concepts, and context) or other relevant key elements used to  conceptualize the review questions and/or objectives. | 2 |
| **METHODS** | | | |
| Protocol and registration | 5 | Indicate whether a review protocol exists; state if and where it can be accessed (e.g., a Web address); and if available, provide registration information, including the registration number. | 2 |
| Eligibility criteria | 6 | Specify characteristics of the sources of evidence used as eligibility criteria (e.g., years considered, language,  and publication status), and provide a rationale. | 3 |
| Information sources* | 7 | Describe all information sources in the search (e.g., databases with dates of coverage and contact with authors to identify additional sources), as well as the date the most recent search was executed. | 2 |
| Search | 8 | Present the full electronic search strategy for at least 1  database, including any limits used, such that it could be repeated. | Appendix |
| Selection of sources of evidence† | 9 | State the process for selecting sources of evidence (i.e., screening and eligibility) included in the scoping review. | 3 |
| Data charting process‡ | 10 | Describe the methods of charting data from the included sources of evidence (e.g., calibrated forms or forms that have been tested by the team before their use, and whether data charting was done independently or in duplicate) and any processes for obtaining and  confirming data from investigators. | 3 |
| Data items | 11 | List and define all variables for which data were sought and any assumptions and simplifications made. | 3 |
| Critical appraisal of individual sources of evidence§ | 12 | If done, provide a rationale for conducting a critical appraisal of included sources of evidence; describe the  methods used and how this information was used in any data synthesis (if appropriate). | Not performed |
| Synthesis of results | 13 | Describe the methods of handling and summarizing the data that were charted. | 3 |
| **RESULTS** | | | |
| Selection of sources of evidence | 14 | Give numbers of sources of evidence screened, assessed for eligibility, and included in the review, with  reasons for exclusions at each stage, ideally using a flow diagram. | 4 |
| Characteristics of sources of evidence | 15 | For each source of evidence, present characteristics for which data were charted and provide the citations. | 12 |
| Critical appraisal within sources of evidence | 16 | If done, present data on critical appraisal of included sources of evidence (see item 12). | Not performed |
| Results of  individual sources of evidence | 17 | For each included source of evidence, present the  relevant data that were charted that relate to the review questions and objectives. | 4 |
| Synthesis of results | 18 | Summarize and/or present the charting results as they relate to the review questions and objectives. | 4 |
| **DISCUSSION** | | | |
| Summary of evidence | 19 | Summarize the main results (including an overview of concepts, themes, and types of evidence available), link to the review questions and objectives, and consider the relevance to key groups. | 7 |
| Limitations | 20 | Discuss the limitations of the scoping review process. | 8 |
| Conclusions | 21 | Provide a general interpretation of the results with respect to the review questions and objectives, as well  as potential implications and/or next steps. | 9 |
| **FUNDING** | | | |
| Funding | 22 | Describe sources of funding for the included sources of evidence, as well as sources of funding for the scoping  review. Describe the role of the funders of the scoping review. | Title page |

**Appendix 2: MEDLINE search strategy 12 May 2023**

| **Search number** | **Search terms** | **Number of results** |
| --- | --- | --- |
| 1 | exp Dementia/ | 203903 |
| 2 | (alzheimer* or dement* or creutzfeldt or lewy bod* or korsakoff* or fronto*tempor* or (vascular adj2 dement*)).tw. | 285433 |
| 3 | 1 or2 | 322589 |
| 4 | ((goal* adj2 care*) or priorit* or needs or requir* or want* or experie* or histor*).tw. | 4867157 |
| 5 | "Health Services Needs and Demand"/ or medically underserved area/ or patient care management/ | 66683 |
| 6 | 4 or 5 | 4904463 |
| 7 | exp consumer health information/ or health communication/ or information dissemination/ or health information management/ or medication therapy management/ | 38439 |
| 8 | (info* or knowledge* or guidan* or advic* or resourc*).tw. | 3103216 |
| 9 | 7 or 8 | 3120806 |
| 10 | (medicat* or drug* or medici* or prescri*).tw. | 2912817 |
| 11 | prescriptions/ or drug prescriptions/ or deprescriptions/ or self administration/ or self medication/ | 53706 |
| 12 | 10 or 11 | 2925740 |
| 13 | 3 and 6 and 9 and 12 | 1910 |

**Appendix 3: PRISMA flowchart**

Studies from databases/registers **(n = 18487)**

References from other sources **(n = 0)**

**Identification**

Studies included in review **(n = 35)**

Studies excluded **(n = 11367)**

Studies not retrieved **(n = 0)**

Studies assessed for eligibility **(n = 149)**

Studies sought for retrieval **(n = 149)**

Studies screened **(n = 11516)**

Studies excluded **(n = 114)**

Not an original study (n = 18)

Not in English (n = 2)

No full-text available (n = 38)

Report researcher priorities. (n = 1)

Not people with dementia or their carers (n = 3)

Report healthcare professional priorities. (n = 3)

Do not report medication management information needs. (n = 49)

References removed **(n = 6971)**

Duplicates identified manually (n = 1763)

Duplicates identified by Covidence (n = 5208)

**Screening**

**Included**
